# Acute Suppuration of the Middle Ear

**Published:** 1886-11

**Authors:** W. Cheatham

**Affiliations:** Louisville, Ky.; Lecturer on Diseases of Eye, Ear, Throat and Nose, University of Louisville; Eye, Ear, Throat and Nose Physician, Louisville City Hospital; Masonic Widows and Orphans’ Home and Infirmary, Etc.


### ACUTE SUPPURATION OF THE MIDDLE EAR.

BY W. CHEATHAM, M. D., LECTURER ON DISEASES OF EYE, EAR, THROAT AND NOSE, UNIVERSITY OF LOUISVILLE; EYE, EAR, THROAT AND NOSE PHYSICIAN, LOUISVILLE CITY HOSPITAL; MASONIC WIDOWS AND ORPHANS' HOME AND INFIRMARY, ETC.

In a previous number of this journal, I endeavored to give the management of "Ear Ache," and how to prevent inflammations of the middle ear going on to suppuration, and especially the supervention of chronic suppuration. Taking up the subject at this point I wish to add chapter No. 2. Suppose, notwithstanding the treatment pursued, suppuration of the middle ear has followed; the patient gives the history of ear ache, with a sudden popping sensation in ear, and immediately afterwards a discharge of muco-pus and blood from external auditory canal. What have we here to deal with? Usually a highly inflamed drum-cavity and drum-membrane, with a large or small perforation of the latter; many of these cases recover by simple cleansing with warm water and absorbent cotton. But many others are not near so fortunate, and get well only after weeks or months of great care and attention. This fall suppurative inflammations of the ear have been exceedingly common, no doubt the result of the numerous colds or catarrhal inflammations of nose and throat now so prevalent. Three weeks ago I was called to see little Miss W., age nine years. I found her with nasal obstruction, the result of an acute nasal catarrh engrafted in a chronic catarrh, with post-nasal hypertrophies and hypertrophy of the third or pharyngeal tonsil. She gave a history of having to sleep with her mouth open, nasal respiration being almost impossible. She had had a severe ear ach, but the ear was now discharging; temperature 102°; extremely nervous with choreic movements. Skin dry and hot, tongue heavily coated. Ear exceedingly sensitive, so much so as to render cleansing it almost impossible. Membrana tympani inflamed, and much thickened, with a perforation, through which pus could be forced when patient performed Valsalva. As the fluid lodged in the perforation, pulsation could be distinctly seen. I prescribed poultices to be applied over mastoid, *not over ear;* the ear to be syringed, by means of the fountain syringe, with hot water every two hours, and wiped thoroughly dry with absorbent cotton; the canal to be plugged with a piece of the cotton afterwards. A calomel and bi-carbonate of soda purge was ordered for bed-time, and sodiumbromide gr. x. after meals to give rest and as a nervine. The next morning I found she had passed a comfortable night, temperature normal, choreic movements much less, and ear discharging freely; this latter I encouraged for several days, then reducing the syringing to four per day, thinning the poultices gradually, finally leaving them off entirely, and substituting a flannel rag, and for this in a few days a linen compress, thus removing the head wraps gradually. All acute trouble having subsided at the end of the sixteenth day, the discharge in a few days ceased and the drum-head healed. The treatment of this case does not end here. All nasal, post-nasal and pharyngeal hypertrophies must be removed. The patient's general condition being below par, she was placed on Flexner's syr. albuminate of iron (which, I must say here, I believe to be the best preparation of iron in the market now). The eustachian catheter and the Politzer bag should be used for some days, or else all labor will be lost, and as soon as the patient is again exposed to slight atmospheric changes, the same trouble will follow and your patient will pass into the hands of, I hope, some one more competent, who, recognizing the cause of the relapse, will treat it as you should have done, and give permanent relief.

Mrs. A., four weeks ago, called me to see her; I found her in somewhat the same condition as little Miss W., only with more pain. She had been unable to sleep for two nights, for which her physician had prescribed an opiate. I found the ear discharging muco-pus with an extremely disagreeable odor. After some difficulty, I succeeded in getting ear clean. The auditory canal was so swollen, just external to the drum-head, that I could not see the condition of the latter. For this case I prescribed three leeches, one to be put on the anterior wall of the external auditory canal and the other two on tragus, to be left on until they dropped off themselves, and to let the bites bleed for half an hour. If this did not relieve the pain, she was to take her opiate. Calling the following afternoon, I found the cotton that had been used to stop the leech bites still in position and a disagreeable odor from the ear pervading the room. The cotton was removed and the ear syringed (fountain syringe) with hot water. The swelling of canal had subsided so a large perforation in lower part of drum-head could be seen distinctly. The patient had had a comfortable night without the opiate. Poultices over mastoid and hot water syringing directed. The inflammation subsided rapidly, and in three weeks the patient was ready for treatment to stop suppuration which had not yet ceased.

Master S., age thirteen, came to me with an intense ear ache. I found membrana tympani bulging and red. I made a puncture which allowed muco-pus and blood to escape. Pain not ceasing, leeches were ordered. For a week all progressed well; when he came to my office I found tenderness over mastoid, with great œdema of the part, pittings on pressure, and pushing auricle forward, or, in other words, getting up a pretty lively mastoid periostitis. With persistent hot-water syringing and poulticing, this soon subsided. Having now no pain, a quite free discharge, poultices were left off, but syringing four times a day continued. The discharge at the end of four weeks continuing, after all acute symptoms had subsided, he was put on the following treatment. Let me lay stress here, though, upon the importance of the subsidence of all acute symptoms, or else the following treatment will produce an exacerbation, and all previous treatment, with its much suffering and often more serious results, will have to be gone through with again. Treatment of suppuration of the middle ear, as many of us know, has undergone many changes in late years, and in some respects for the better, yet we occasionally find cases which can be cured only by a return to the old ear drops of argent. nitrate, zinc sulpho-carbolate, etc. That now pursued by many may be called the dry treatment. I myself have endeavored to strike a compromise between the new and the old. I recognize no method of cleansing the ear equal to the syringe, warm soda water and the peroxide of hydrogen. I direct that the ear be washed out as often as necessary with warm water, with two teaspoonsfuls of soda bi-carb to the pint; wipe as dry as possible with absorbent cotton. Let patient hold head to one side and pour in eight or ten drops of peroxide of hydrogen. A bubbling and stirring will follow and all pus forced from every recess. This is now to be wiped out and a few drops of alcohol, 40 per cent., 50 per cent. or 60 per cent., or as strong as the patient will bear; put in and left there for several minutes and again wiped out; this leaves the ear thoroughly dry, and has corrected all damage that can be attributed to a careful syringing with water, and I claim is ahead of all other methods of cleansing, which all of us recognize is the one above all the necessaries in the successful management of acute suppuration of the middle ear. The alcohol may be used weak in the beginning, and its strength gradually increased as the ear becomes accustomed to it. The ear now being thoroughly cleansed and dried, it is to be well packed with some antiseptic and inocuous powder, the three following being the best, and given as I prefer them: First, finely pulverized boracic acid, iodol and oxide of zinc. They may be used either separate or combined. I don't believe simply blowing them in is of much service. I direct that they be packed in from the bottom, and each night and morning fresh powder be put on top of the old, and again pressed down, the ear to be cleansed every one, two or three days, or once a week, or just as often as thought best. Another method I have of using these powders is to combine one or all of them with the alcohol, putting ʒ ii. or more of one, two or three of them to ℥ i. of the alcohol, directing that after the ear is cleansed the head is to be held to one side, the bottle well shaken, and the canal half filled with the mixture, the head to be held in this position until most of the alcohol evaporates, leaving the powder well applied to the ear, the alcohol carrying it to all parts of its suppurating surface. This is quite a favorite treatment with me, and yields good results when all others fail. Again the powder can be packed in as before after the alcohol has evaporated. One objection I urge to the packing treatment is that to me it appears quite possible that the powder so packed, unless the suppuration is free enough to soon dissolve it, might prevent the perforation of the membrana tympani from healing by acting as a foreign substance between its edges. The treatment just given relieves a majority of the cases of acute suppuration of middle ear, unless there be some unusual results, such as caries of bone, or large granulations present. The alcohol and powders will get rid of all small granulations and many large ones. When bone caries is present it passes over into chronic suppuration which must be considered later. As I stated a few moments ago, some few cases have to be treated as of old with solutions of nitrate of silver, zinc, etc.

